# New Books

**Published:** 2009-01

**Aerosol Pollution Impact on Precipitation**

Zev Levin, William R. Cotton, eds.

New York:Springer, 2009. 386 pp. ISBN: 978-1-4020-8689-2, $229

**Comparative Genomics**

Craig Nelson; Stéphane Vialette, eds.

New York:Springer, 2008. 265 p. ISBN: 978-3-540-87988-6, $69.95

**Computational Methods in Systems Biology**

Monika Heiner, Adelinde M. Uhrmacher, eds.

New York:Springer, 2008. 403 pp. ISBN: 978-3-540-88561-0, $89.95

**Coral Bleaching: Patterns, Processes, Causes and Consequences**

Madeleine J. H. van Oppen, Janice M. Lough, eds.

New York:Springer, 2009. 178 pp. ISBN: 978-3-540-69774-9, $119

**Creating Scientific Concepts**

Nancy Nersessian

Cambridge, MA:MIT Press, 2008. 272 pp. ISBN: 978-0-262-14105-5, $32

**Encyclopedia of Paleoclimatology and Ancient Environments**

Vivien Gornitz, ed.

New York:Springer, 2009. 1,049 pp. ISBN: 978-1-4020-4551-6, $499

**Global Climatology and Ecodynamics: Anthropogenic Changes to Planet Earth**

Arthur Philip Cracknell, Vladimir F. Krapivin, Costas Varotsos

New York:Springer, 2008. 520 pp. ISBN: 978-3-540-78208-7, $259

**Institutions and Environmental Change: Principal Findings, Applications, and Research Frontiers**

Oran R. Young, Leslie A. King, Heike Schroeder, eds.

Cambridge, MA:MIT Press, 2008. 400 pp. ISBN: 978-0-262-24057-4, $70

**New Directions in Climate Change Vulnerability, Impacts, and Adaptation Assessment**

Jennifer F. Brewer

Washington, DC:National Academies Press, 2008. 40 pp. ISBN: 978-0-309-13006-6, $15

**Reviews of Environmental Contamination and Toxicology, Vol. 196**

David M. Whitacre, ed.

New York:Springer, 2009. 205 pp. ISBN: 978-0-387-78443-4, $109

**Reviews of Environmental Contamination, Vol. 197: Arsenic Pollution and Remediation**

Hemda Garelick, Huw Jones, eds.

New York:Springer, 2008. 194 pp. ISBN: 978-0-387-79283-5, $139

**Science and Decisions: Advancing Risk Assessment**

Committee on Improving Risk Analysis Approaches Used by the U.S. EPA, National Research Council

Washington, DC: National Academies Press, 2008. 478 pp. ISBN: 978-0-309-12046-3, $44.06

**Scientific Collaboration on the Internet**

Gary M. Olson, Ann Zimmerman, Nathan Bos, eds.

Cambridge, MA:MIT Press, 2008. 432 pp. ISBN: 978-0-262-15120-7, $45

**Statistical Methods for Environmental Epidemiology with R: A Case Study in Air Pollution and Health**

Roger D. Peng, Francesca Dominici

New York:Springer, 2008. 144 pp. ISBN: 978-0-387-78166-2, $49.95

**Strategic Bargaining and Cooperation in Greenhouse Gas Mitigations: An Integrated Assessment Modeling Approach**

Zili Yang

Cambridge, MA:MIT Press, 2008. 216 pp. ISBN: 978-0-262-24054-3, $40

**Sustaining Life: How Human Health Depends on Biodiversity**

Eric Chivian, Aaron Bernstein, eds.

New York:Oxford University Press, 2008. 542 pp. ISBN: 978-0-19-517509-7, $34.95

**Technology and Society: Building Our Sociotechnical Future**

Deborah G. Johnson, Jameson M. Wetmore, eds.

Cambridge, MA:MIT Press, 2008. 648 pp. ISBN: 978-0-262-60073-6, $42

